# Carbon markets: a new form of colonialism for Indigenous Peoples?

**DOI:** 10.1016/S2542-5196(25)00086-5

**Published:** 2025-05-14

**Authors:** Nicole Redvers, Josh Chan, Siwakorn Odochao, Victoria Pratt, Jessica Sim, Samrawit Gougsa, Daniel M Kobei, Liz Willetts

**Affiliations:** a Schulich School of Medicine and Dentistry, University of Western Ontario, London, ON, Canada; b Arctic Indigenous Wellness Foundation, Yellowknife, NT, Canada; c Lazy Man Coffee, Chiang Mai, Thailand; d The Hub at Wellcome Collection, London, UK; e Invisible Flock, West Bretton, UK; f Minority Rights Group, London, UK; g Ogiek Peoples’ Development Program, Egerton, Kenya; h Human Health Thematic Group, Commission on Ecosystem Management, International Union for Conservation of Nature, Gland, Switzerland

## Abstract

The interconnected and compounding climate change and biodiversity crises have led to increased urgency in moving towards transformational change within how national and international sustainability efforts are viewed and operationalised. Despite the known benefit of carbon markets as part of these sustainability efforts, there has been increasing scrutiny of carbon market mechanisms, with warranted distrust present at the community level. Indigenous Peoples are key stewards of biodiverse landscapes, yet their exclusion within carbon market decision making is ongoing. With this exclusion, outstanding questions remain on the placement of Indigenous Peoples within current carbon market design and decision making and their roles have yet to be fully appreciated in wider policy and practice. Platformed on substantial inequities, marginalisation, and racism, we therefore query in this Personal View, are carbon markets a new form of colonialism? We further reflect on the challenges and the potential opportunities of carbon markets for Indigenous Peoples and anchor our reflections with examples from different regions.

## Introduction

The co-occurring and interconnected climate change and biodiversity crises have led to more urgency in moving towards transformational change within how national and international sustainability efforts are viewed and operationalised. The surging interest in carbon offsetting and marketable carbon credit permits (panel 1) is projected to lead to the growth of the voluntary carbon-offset market from US$2 billion in 2020 to approximately $250 billion by 2050.^3^ Carbon pricing mechanisms aim, in theory, to help shift the burden for the damage from greenhouse gas emissions back to those who are responsible for it (eg, individuals, organisations, and industries).^4^ Approximately 70 national and subnational governments have introduced a price on carbon pollution,^5^ with a 2024 review noting “consistent evidence that carbon pricing policies have caused emissions reductions…with immediate and sustained reductions of between –5% to –21% (–4% to –15% when correcting for publication bias)”.^6^

Panel 1 **Key terms within carbon markets**

**Carbon credits**

“Carbon credits are marketable permits that each reflect one metric ton of carbon dioxide (CO_2_) emissions (or other greenhouse gases) that a business is allowed to emit. Carbon credits are commonly used in the context of emissions trading in which companies are given a fixed amount of credits depending on their emissions. They can later purchase more credits or sell their extra[s]…Carbon credits are a measurement unit to ‘cap’ emissions (meaning permitted emissions).”^1^

**Carbon offsets**

“Carbon offsets are typically created when companies or individuals finance projects that reduce greenhouse gas emissions elsewhere. Projects to reduce carbon often fall into one of two categories: mechanical or natural…Carbon offsets can be considered a measurement unit to ‘compensate’ a business for investing in green projects or initiatives (whether natural or mechanical) that eliminate emissions.”^1^

**Cap-and-trade system**

“A cap-and-trade system establishes a cap on maximum emissions in order to reduce aggregate emissions from a group of emitters. This market-based approach promotes lower pollutant emissions and promotes investment in energy efficiency and fossil fuel alternatives.”^1^

**Nature-based solutions**

“Nature-based solutions to climate change [mitigation] use plants to draw carbon dioxide from the atmosphere, either by preserving and managing existing ecosystems or by creating new carbon sinks to sequester additional carbon from the atmosphere.”^2^

**Types of nature-based solutions**

For example, avoid deforestation, reforestation, afforestation, managing forests, wetland and peatland preservation, wetland and peatland restoration, agroforestry, and soil carbon sequestration.

**Technological offsets**

The use of technologies to remove and reduce carbon.

Despite the known benefit of carbon markets with regard to reducing emissions, these voluntary carbon market mechanisms have incurred increasing scrutiny due to the scarcity of appropriate safeguards being put in place.^7^, ^8^, ^9^ Whether or not carbon markets themselves serve as creative new modes of accumulation that are unlikely to transform capitalist dynamics in ways that might foster a more sustainable global economy has also been questioned.^7^ In addition, several persistent issues exist in some regions of the world, including the issue of phantom credits (ie, when projects overstate their greenhouse gas emission reductions),^10^ the inadequate overall focus on ensuring that initiatives reduce rather than offset emissions,^11^ inconsistent involvement of local communities in the participation and decision-making processes of mechanisms, and the lack of core focus on cobenefits for local communities. Warranted community-level distrust therefore surrounds the current structure and operations within which carbon markets have been designed and implemented. The urgency of the converging crises that humanity faces within the context of declining planetary health should not supersede equitable practices, yet ongoing human rights violations are commonly accepted as the cost of doing business.^12^, ^13^

Carbon markets are complex and imperfect mechanisms with more attention needed on identifying and determining acceptable social costs, if any, within public and private sectors. Stakeholder participation in these mechanisms is an area of weakness—for example, with decision-making processes for the development and implementation of carbon markets not generally including Indigenous Peoples.^14^ Indigenous Peoples are key stewards of biodiverse landscapes and planetary health, yet their exclusion from many decision-making arenas regarding national and international climate change and biodiversity is ongoing. This exclusion is a continuing consequence of entrenched high-income country versus low-income and middle-income country power dynamics and colonialism. Many outstanding questions remain for the placement of Indigenous Peoples within current carbon markets, and their roles have yet to be fully appreciated in wider policy and practice.

Platformed on substantial inequities, marginalisation, and racism, in this Personal View, we examine whether carbon markets are a new form of colonialism for Indigenous Peoples. We further consider the challenges and the potential opportunities of carbon markets for Indigenous Peoples, using examples from different legal and political contexts. Overall, we aim to provide insights from Indigenous Peoples into the topic of carbon markets that could diverge from current national and international policy discourse.

Given the importance of positionality in writing with or about Indigenous Peoples’ lived experience, we first position ourselves here as a group of Indigenous Peoples (NR, SO, and DMK) from Canada, Thailand, and Kenya; and a group of non-Indigenous allies supporting the work (JC, VP, JS, SG, and LW) from Canada, the UK, and the USA.

## Indigenous Peoples’ rights violations and carbon markets

There have been notable challenges, including rights violations, for Indigenous Peoples working with or against carbon markets and related mechanisms worldwide (panel 2). Foremost are the substantial geopolitical differences in the level of recognition afforded to Indigenous Peoples by national governments. This inconsistent recognition of Indigenous Peoples worldwide leads to a lack of Indigenous leadership or involvement in discussions, design, and the implementation of carbon markets. Additionally, when Indigenous Peoples are not recognised as Indigenous Peoples by national governments and state actors, having a voice or decision-making power in regard to Indigenous lands is difficult, if not impossible. Without political recognition of Indigenous Peoples within a respective region, there is often then an absence of Indigenous land tenure rights, which puts Indigenous Peoples at substantial risk of forced land eviction to make room for conservation efforts. For example, in November, 2023, Ogiek Indigenous Peoples from Kenya were forcibly removed from their traditional territories under a conservation agenda meant to make way for carbon credits.^15^ Carbon-credit projects have the potential to therefore interfere with Indigenous communities’ rights to their own land, affect their use of resources, and affect their ability to pass on traditional ecological knowledges, as well as impact their territorial governance or rights—while additionally displacing communities and impacting their livelihoods.^16^

Panel 2 **Challenges for Indigenous Peoples in relation to engagement with carbon markets**

• Little to no recognition of Indigenous Peoples in many countries
• Lack of Indigenous land tenure rights in many countries
• Insufficient decision-making power for Indigenous Peoples regarding their lands
• Little to no capacity and inadequate availability of technical resources on carbon markets within Indigenous communities
• Unaddressed language barriers relevant for communications, decision making, and informed consent
• Little to no awareness and respect for free, prior, and informed consent
• Blatant exploitation from state and corporate actors impacts trust and relationship building
• State or corporate actors disregarding already agreed-upon community-based carbon market terms of agreements, with no consequences
• Patriarchal and colonial approaches to community engagement and policy creation (eg, top-down, high-income country, and corporate domination of agenda setting, funding arrangements, and decision making centring those other than Indigenous communities)
• Direct Indigenous rights violations for Indigenous Peoples, forced displacement, and conflict stemming from projects related to carbon mitigation (eg, conservation activities)
• The Eurocentric transactional view of the land as a resource or commodity to serve humans (ie, natural capital, ecosystem service, or nature's contribution to people)

Low capacity and inadequate technical resources, as well as inequitable benefit distribution, additionally hinder the participation of Indigenous Peoples in carbon markets.^17^ Carbon markets and the paperwork that comes with them (eg, offsetting deals) are often technically complex and difficult to navigate for non-experts. Indigenous language barriers add complexity, putting Indigenous Peoples at substantial risk for exploitation and marginalisation. In turn, carbon market design teams also then fail to incorporate Indigenous knowledges due to the disregard of the importance of language. Indigenous communities also face increasing threats from so-called carbon pirates and unfair agreements (ie, biased agreements towards corporate actor benefits over Indigenous communities), leading to land dispossession and unfulfilled financial promises.^13^ Weak regulation in carbon market mechanisms and a lack of transparency exacerbate these issues where anticipated or agreed upon financial benefits sometimes do not end up reaching Indigenous communities.^18^ Blatant exploitation often leads to conflict between communities and project developers or within communities, with rights violations, displacement, and conflict often stemming from failure to obtain the full consent of communities or disregarding local governance practices.^16^ These negative experiences with carbon markets can also have the counter effect of decreasing climate change resilience within communities, while exacerbating negative health impacts and loss and damage from concurrent global environmental changes.^19^

Direct violations to Indigenous Peoples’ free, prior, and informed consent with carbon-related projects, or blatant disregard of valid carbon-related agreements with Indigenous Peoples have been increasingly documented.^14^ These violations lead to valid scepticism and outright rejection of carbon-related projects within some Indigenous territories.^13^, ^20^, ^21^, ^22^, ^23^ Negative experiences have also led to the development of some toolkits and resources to support Indigenous Peoples in relating with outside entities for carbon-related projects on Indigenous lands.^24^, ^25^ Regardless, available resources and support for Indigenous Peoples to navigate the complexities of carbon-based mechanisms are scarce in many regions.

At a fundamental level, within many Indigenous communities, there is often a strong rejection of the carbon market concept existing that has not been effectively appreciated or addressed. For example, many Indigenous Peoples have great difficulty putting a monetary value on their lands and waters. Indigenous lands and water are often seen to be living relatives, not something to be owned or traded.^26^ Therefore, the fundamental values underpinning carbon markets (or the lack thereof) are often in direct opposition to how Indigenous Peoples relate to the land, creating ethical dilemmas that are not often appreciated by outside entities. From an Indigenous relational worldview, the Eurocentric view of the land as a resource or commodity and an “ideology of independence has resulted in a sense of entitled ownership, a kind of utilitarian perception of the natural world that relates to it through transactional relationships that do not have a sense of responsibility, care, or love”.^27^ This Eurocentric worldview of humans being disconnected from the land they walk on^28^, ^29^ perpetuates climate solutions (eg, carbon markets) that are also, themselves, devoid of relational connections to the land. It has been stated that “[w]e cannot solve complex problems from the same worldview that created them in the first place, as it will continue to perpetuate a disconnect between us and the planet as ‘relatives’.”^30^ Carbon markets derived from colonial systems therefore diverge conceptually from how many Indigenous communities relate to their landscapes. Along these lines, from an Indigenous perspective,

“…[h]umans have [in many cases] lost their identity as organisms within a larger system and thus have lost awareness of how to live sustainably with Mother Earth. Ecological demise points to an impaired human relationship with its inner self (ie, humans are Nature and not apart from it). In the broader sense, there is evidence of the loss of an ecologically bound cultural identity. The disconnect from Nature manifests as a fragmented and dissociated identity that cannot recognise itself as part of a system, making it easier to project predatory and abusive impulses onto the environment.”^27^

Overall, with Indigenous worldviews often differing substantially from Eurocentric worldviews,^28^, ^29^ there is a fundamental incongruence between how carbon markets have been formulated to value the land compared with how Indigenous Peoples relate with the land. We present an on-the-ground case example from Thailand to further highlight the challenges for Indigenous Peoples related to engagement with carbon markets.

### Case example: Indigenous Peoples in Thailand

The Kunming-Montreal Global Biodiversity Framework has 23 action-oriented global targets for urgent action, over the decade, to 2030^31^ and includes the common reference to the 30 × 30 target (ie, the protection and management of 30% of the world's terrestrial, inland water, and coastal and marine areas being effectively conserved and managed or restored by the year 2030).^32^ This target is an addition to other global initiatives, such as the Glasgow Leaders’ Declaration on Forests and Land Use, which countries such as Thailand have signed with the commitment to raise Thailand's forest cover to 55% of the country's total area, and achieve net zero emissions and carbon neutrality.^33^ Market-based conservation agendas are codeveloping alongside international biodiversity protection movements, often perpetuating so-called fortress conservation approaches (ie, a conservation approach that separates people from their landscapes) in some regions, particularly in some low-income and middle-income countries.^34^ This approach has led to the false narrative (explicit or implicit), often amplified through policy and discourse, that to effectively conserve biodiversity, there can be no Indigenous Peoples in the conserved areas. This idea has led to violent conflicts, forced land evictions, and an increasing number of Indigenous conservation refugees worldwide,^35^, ^36^ including in Thailand.^20^ It has also led to ongoing tensions around the concept of conservation in global governance.^37^

In Thailand, carbon credit policies and initiatives are at a turning point. There is a current blend of ambitious projects and the acknowledgment of substantial challenges existing in the country. The overall implementation of carbon credits aims to promote clean energy and reduce emissions, yet it faces increasing scrutiny in Thailand regarding its effectiveness and integrity.^14^ Allegations of inflated and fabricated figures for emission reduction have cast doubt on the legitimacy of carbon-related projects, prompting calls for stricter regulation and greater transparency.^23^ The lack of accountability in the verification and certification processes has further exacerbated concerns, potentially harming the country's reputation and undermining genuine climate action efforts.^38^ The introduction of Thailand's new clean energy and carbon credit trading platform is a key step towards bolstering the national carbon market. Operating voluntarily and without government regulation, this platform is designed to facilitate Thai exporters in meeting international carbon emission standards.^38^ Despite its potential to enhance Thailand's climate mitigation efforts, the unregulated nature of the platform raises questions about the long-term sustainability and effectiveness of such initiatives.^23^

Thailand's engagement with carbon credits as a strategy for forest conservation and climate change mitigation has had substantial implications for Indigenous Peoples in the region. This situation is further complicated by the fact that Thailand does not recognise its own Indigenous Peoples,^39^ and therefore no formal complaints or adjudication body to address land or human rights issues specific to Indigenous Peoples exists in Thailand. The Government of Thailand's Reducing Emissions from Deforestation and Forest Degradation programme aims to incentivise the reduction of deforestation while promoting sustainable forest management;^40^ however, the implementation of carbon credit policies has led to the restriction of Indigenous Peoples’ access to forests and land, resulting in their marginalisation and displacement. Traditional communities face forced relocations and conflicts, with state and corporate interests often being prioritised over communities’ rights and knowledges.^23^ These experiences in Thailand highlight the need for more inclusive and rights-based approaches that platform Indigenous rights and promote community-based conservation efforts.

Additionally, the expansion of the carbon credit market in Thailand and its implications for Indigenous lands require careful consideration to avoid perpetuating ongoing social injustices. Instances of exploitation and inadequate consultation underscore the need for strict regulations and genuine engagement with Indigenous Peoples and their communities.^14^ The current situation in the north of Thailand, for example, highlights the complexity of Indigenous land management, especially when examined through the lens of carbon markets.

### Conservation, carbon markets, and the Ban Nong Tao and Huay Ee Khang Indigenous Peoples

In Thailand, the National Land Policy Board is using satellite assessments^41^ to re-categorise lands into four zone types (based on land use since 2002),^42^, ^43^ thus threatening Indigenous communities’ traditional farming practices. A law passed in 2014 (Ko To Cho คทช), has since mandated that, depending on the zone, between 20% and 70% of land must remain forested. However, this new land categorisation is causing confusion within Indigenous communities in Thailand. For example, families in Karen communities, such as Pgak'yau of Ban Nong Tao and Huay Ee Khang, struggle to understand the implications of a land-use permit, which in the Chiang Mai province (where they are located), is granted for 30 years.^44^ The land lease, although permitting the community to legally farm in certain areas,^41^ does not provide official land titles, further limiting how the land can be used. There is also concern that this government land lease system will make it difficult or impossible for Indigenous Peoples to claim full land rights in the future.

Additionally, the government land lease system does not consider the Indigenous and sustainable rotational farming practices that have been done for generations in the region, especially for land with more than a 30% inclined slope (key land for Indigenous traditional farming practices). The Forestry Department in Thailand does not recognise that farming practices can take place on mountain slopes,^45^ and as such, these areas have been categorised as conservation forest (ie, land that cannot be used by Indigenous Peoples for traditional farming). Traditionally, Pgak'yau have always farmed lowland and mountain areas, rotating to different land plots so that the same piece of land is not used for more than 7 years, to allow the soil and plants to regenerate. However, current government land policy contradicts the ecological stewardship approach of Indigenous land use. Many Indigenous farmers now feel pressed to over-farm these re-categorised plots, clearing them every 2–3 years instead of the traditional 7 years, to avoid the Forestry Department reclassifying their plots as conservation forest, and consequently losing their land. This Indigenous community is therefore forced by government conservation policy to abandon planetary stewardship, a practice that, ironically, has many positive impacts on long-term soil health and biodiversity in the region.^46^

In addition to biodiversity concerns, there is also confusion about how these new government land policies intersect with carbon credit schemes. The Forest Carbon Credit Management Project for Sustainable Development in Thailand^47^ provides a model for fostering collaboration between local communities and the private sector to enhance forest conservation while reducing carbon emissions. Through this project, communities gain financial support for sustainable land management, while private sectors receive carbon credits, which promotes Thailand's efforts towards carbon neutrality. In areas of Thailand, such as Mae Chaem, monoculture farming dominates the landscape; however, not all Indigenous regions use this method; some traditionally have thrived on sustainable rotational farming. Indigenous communities, such as the Pgak'yau, argue that the current carbon credit structure in Thailand is more suited to large-scale monoculture, and not to the diverse Indigenous practices including rotational farming on mountain slopes. For Pgak'yau, carbon credit schemes are often seen to go against their fundamental beliefs and culture about how to live in balance with nature, seeing the land as not something that can be owned. As local communities advocate for land rights and more sustainable farming alternatives, the challenge remains to integrate these efforts with broader policies, ensuring that Indigenous knowledges and practices are recognised and supported.

Broader conservation policies in Thailand link back to 2021 when the country pledged at the UN Framework Convention on Climate Change (UNFCCC) 26th Conference of the Parties (COP) to increase forest cover to at least 55% by 2030.^33^ However, this pledge, although seemingly beneficial for the environment, risks marginalising Indigenous communities in the region who have cared for their land sustainably for generations. Without acknowledging Indigenous Peoples and their knowledge systems and land management practices, Thailand's national policies are doing more harm than good, creating a one-size-fits-all solution that fails to consider local contexts and Indigenous rights. The complex land-use systems in mountain Indigenous communities in Thailand, which include managed forests and shifting cultivation, provide crucial ecological services in carbon storage and highlight the substantial role of Indigenous Peoples and their knowledges in sustaining both livelihoods and the environment.

In 2024, Thailand's Ministry of Natural Resources and Environment launched a carbon credit exchange for 121 community forests, aiming to support sustainable forest management as part of Thailand's carbon neutrality goal by 2050.^48^ Although the carbon market could offer a potential revenue stream for Indigenous communities in the region, a new marketplace is needed that prioritises Indigenous values and incorporates Indigenous indicators of land management. A new marketplace is particularly important for communities, such as the Pgak'yau of Ban Nong Tao, as although Thailand is a signatory of the UN Declaration on the Rights of Indigenous Peoples, Indigenous Peoples are not officially recognised by the Thai Government. In 2024, the National Assembly of Thailand voted to remove the words Indigenous People from Thailand's first ethnic rights bill.^39^ Indigenous Peoples in Thailand therefore remain unprotected by the UN Declaration on the Rights of Indigenous Peoples or other Indigenous rights mechanisms and are subjected to land management policies without consent or mechanisms to contest them. If carbon crediting is to continue to take root in Thailand, there is a need to cocreate a system that generates income for Indigenous communities without eroding the cultural and environmental integrity of Indigenous lands.

## Potential opportunities with carbon markets for Indigenous Peoples

Ensuring that carbon credit schemes avoid exacerbating existing inequalities and do not violate Indigenous rights, while supporting the self-determination and economic self-sufficiency of Indigenous Peoples, is crucial for achieving both environmental and social justice in climate change mitigation and conservation efforts. There are increasing carbon market opportunities for interested Indigenous nations, which could lead to economic benefits (eg, economic self-sufficiency), and a potentially increased ability to protect some land bases despite drawbacks (panel 3).^49^ Economic benefits for Indigenous Peoples from traditional land management practices have been consistently cited as a potential incentive for Indigenous participation in such carbon market activities. These programmes could also help to preserve intergenerational knowledge and traditional land stewardship practices through funded mechanisms led by Indigenous Peoples. Despite these potential benefits, based on the many challenges noted in panel 2 (eg, Indigenous rights violations, forced displacement, and an absence of free, prior, and informed consent), ensuring that Indigenous communities are key leaders in any decision-making processes is crucial, while also ensuring that free, prior, and informed consent is a requirement for outside entities. Additionally, ensuring interested Indigenous nations are fairly compensated through appropriate and rights-based cobenefit agreements is paramount.^14^, ^40^

Panel 3 **Potential opportunities for Indigenous Peoples who are interested in engagement with carbon markets (particularly markets that are Indigenous-led)**

• Economic benefits including improving economic self-sufficiency and maintaining livelihoods
• Potential opportunity to preserve key traditional territories
• Preservation of traditional land management practices through funded mechanisms
• Where enabled, greater respect and inclusion of Indigenous knowledges
• Where enabled, greater decision-making power for intergenerational land stewardshipWhere enabled, Indigenous leadership in conservation strategies
• Capacity building within Indigenous communities
• Supporting land management activities that support existing environmental and cultural and enterprises^17^
• Maintaining or improving the health of the land

The importance of stringent regulation to hold governments and corporate entities to account with carbon market initiatives is essential. Additionally, the upholding of human and Indigenous rights, and the respect for genuine participation and decision making, needs to be firmly underscored to restore credibility and better ensure the success of carbon-offset initiatives within interested Indigenous territories. Some Indigenous leaders have advocated for equitable benefit distribution, respect for Indigenous knowledges, and appropriate and respectful consultation in carbon-offset projects. Meaningful engagement and capacity building for Indigenous communities to be better able to navigate carbon market complexities successfully and ensure projects align with their values and rights are sorely needed.^17^, ^50^ For instance, the case of Kalpowar Station in Australia highlights how traditional owners can negotiate fairer carbon credit agreements, ensuring their rights and knowledge are respected, while also benefiting economically from sustainable land management practices.^51^ Additionally, in Australia, Indigenous-led carbon credit projects emphasise the value of traditional knowledges and environmental stewardship, with efforts focused on both economic gain and sustainable land management (panel 4).^51^

Panel 4 **Conservation, carbon markets, and Aboriginal Peoples in Australia**

The Carbon Farming Initiative enables Aboriginal communities in Australia to leverage traditional knowledges in carbon-offset projects. These projects inform practices that enhance biodiversity alongside carbon sequestration.^52^ They reflect a holistic approach to land management that prioritises both carbon mitigation and cultural goals. In southern and eastern Australia, successful projects contribute to carbon mitigation and restore Indigenous stewardship over traditional lands. Many of these projects involve planting trees in areas that are identified as suitable for high carbon sequestration.^17^ These projects have been instrumental in fostering economic opportunities and restoring Aboriginal Peoples’ stewardship over traditional lands in the region.^17^ By integrating Indigenous knowledges and practices, carbon projects in the region have promoted intercultural exchanges and supported socioeconomic development for Aboriginal communities.^53^ The projects have additionally provided avenues for local participation in managing commercial activities and re-establishing a connection with their lands.^53^

Australia's carbon credit programme has also been implemented to address wildfire management while additionally providing socioeconomic and environmental benefits for Aboriginal communities. The programmes have led to better fire management outcomes, including reduced dry season wildfires and increased early season prescribed burns, which has been shown to reduce greenhouse gas emissions.^54^, ^55^ As of 2021, 32 Aboriginal-owned and operated savanna fire projects were underway across 17·9 million hectares in northern Australia, with their work having abated around 1 million tonnes of emissions per year, and having generated around AUD$95 million in Australian Carbon Credit Units since 2012.^56^

Aboriginal communities in the country have benefited from the Indigenous-led carbon credit programme through financial gains, enhanced community engagement in fire management practices, and improved social and ecological outcomes. Additionally, the carbon credit programme has shown potential for positive biodiversity outcomes by maintaining fire-vulnerable taxa and improving ecosystem health.^54^, ^57^ Overall, Australia's carbon credit landscape, although regionally based, has provided a promising framework for integrating Indigenous-led fire management with climate change mitigation efforts, yielding direct benefits to communities. However, there is a continued need to “understand the process of generating verifiable carbon credits to sustain these Indigenous fire management programs” in other regions of the world.^55^ Regardless, integrating Aboriginal Peoples into Australia's carbon markets has been seen to help foster a sustainable future that benefits both the environment and self-determination of Indigenous communities.

Various carbon credit agreements and initiatives have also been developed to assist Indigenous communities economically. For example, in British Columbia, Canada, Indigenous communities are leveraging carbon credits to protect their lands, foster economic independence, and combat climate change. The Atmospheric Benefit Sharing Agreement in British Columbia allows Coastal First Nations to own and sell carbon offsets, thus reducing industrial logging and generating revenue.^58^ Although there are important nuances that create jurisdictional complexities on so-called crown lands, the benefit sharing agreement is a start for “First Nations to negotiate carbon rights on a government-to-government basis through negotiated treaties and reconciliation agreements”.^49^

Similarly, the agreement between Mosaic Forest Management and Indigenous communities in British Columbia defers logging and allows substantial earnings through carbon credit sales. This project showcases the potential for Indigenous rights recognition and profitability in forest preservation.^59^ Specifically, Mosaic Forest Management's 25-year deferral of logging on 100 000 acres of land in Vancouver Island and Haida Gwaii offers Indigenous Peoples up to CA$300 million from carbon credits. By supporting Indigenous rights, land management, and environmental stewardship, this approach has the potential to strengthen conservation, provide Indigenous communities with a source of revenue, and reduce atmospheric carbon.

## Multilateral environmental agreements

Several national and international platforms and advocacy efforts have emerged to advance carbon market mechanisms. Multilateral environmental agreements for both climate change and biodiversity continue to consider and operationalise elements of this work, and have been said to have made some advancement in social safeguards in 2024. However, from an Indigenous perspective these stated social safeguards are not often seen to be adequate for appropriate community protections, as outlined in this Personal View.

Under the UNFCCC, a crediting mechanism for voluntary cooperation and financing was developed through the Paris Agreement (Article 6).^60^ The mechanism essentially allows countries to transfer carbon credits earned from the reduction of greenhouse gas emissions to help one or more countries meet their climate targets. The mechanism is intended to guide progress and its rules, modalities, and procedures (Article 6.4) with appropriate controls and safeguards. However, at UNFCCC COP29 the process advanced without clear standards on social and environmental safeguards, leaving room for weak consideration of human and Indigenous rights violations.^61^ A decision was also taken without clear standards on methodology, such as for equitable sharing of benefits and for broad stakeholder participation.^62^ The process also advanced without clear standards on removals, which includes “avoidance of other negative environmental and social impacts and respecting human rights and the rights of Indigenous Peoples”.^62^

These concerns were amplified during the UNFCCC COP29 by the International Indian Treaty Council, who joined with other participants of the International Indigenous Peoples Forum on Climate Change (representing over 150 Indigenous Peoples worldwide) warning that these market-based mechanisms threaten Indigenous Peoples’ ecosystems and rights, and enable continued pollution under the guise of climate action.^63^ This Indigenous coalition has asked for recognition of Indigenous “time-tested methods and practices for ecosystem protection, restoration and resiliency to address and minimize the climate crisis…[instead of] the fast-tracking of carbon market schemes that allow states to evade accountability while putting Indigenous Peoples’ health, safety, and rights at risk”.^63^

1 month before COP29, the Convention on Biological Diversity (CBD) adopted a decision on Biodiversity and Climate Change at CBD COP16 in 2024 that urged parties to implement effective social and environmental safeguards that can be used in the design of these mechanisms (panel 5).^64^

Panel 5 **Environmental and social safeguards in the Convention on Biological Diversity decision 16/22 (Biodiversity and Climate Change)64 adopted in 2024**

• To identify synergies between biodiversity and climate change for the full carbon cycle
• To promote positive impacts and minimise negative impacts of climate actions on biodiversity and ecosystem integrity, particularly for Indigenous Peoples and local communities that depend on biodiversity
• To consider integrating and promoting nature-based solutions or ecosystem-based approaches, non-market-based approaches, and Mother Earth-centric actions, as recognised by some countries, to climate change adaptation, mitigation and disaster risk reduction, and relevant national plans under various multilateral environmental agreements (ie, Convention on Biological Diversity and UN Framework Convention on Climate Change)
• Inviting the UN Framework Convention on Climate Change and its parties to consider use of the Convention on Biological Diversity's voluntary guidelines^65^ on ecosystem-based approaches to climate change to integrate biodiversity and social safeguards in mitigation and adaptation measures
• To take into account the diversity of values, worldviews, and knowledge systems, including traditional knowledge of Indigenous Peoples and local communities, and the intersectional approaches to ensure contextually relevant actions for respecting, protecting, promoting, and fulfilling human rights and enhancing empowerment, agency, and intergenerational equity and the protection of Indigenous Peoples’ and local communities’ rights over their lands, territories, and resources, and that potential synergies between biodiversity and climate actions that have a direct or indirect impact on land rights or human rights, as well as the rights of Indigenous Peoples and local communities, should only be undertaken with their free, prior, and informed consent

This newly adopted CBD decision took several years to reach consensus, and the success of its implementation will depend on concerted and integrated action at the national level. It will also be most effective if it is used as a tool by the UNFCCC and other multilateral environmental agreements. The use of the CBD decision can be reinforced by a new platform for participation, also adopted at CBD COP16, the new subsidiary body for engagement of Indigenous peoples and local communities (Subsidiary Body on Article 8j).^66^ The new body intends to provide a formal and permanent role to Indigenous Peoples and local communities, and for traditional and local knowledges to influence global biodiversity governance.^67^ The agreement to establish a new Indigenous Peoples caucus at the UN Convention to Combat Desertification COP16 in December, 2024, is also a step in the right direction for inclusive global governance of land.^68^ However, the operation and ability of these new bodies to implement key change within international structures is yet to be seen. However, no such body exists for the UNFCCC COP processes, which is a crucial gap moving forward in the climate change area and for effectively and equitably linking climate change and conservation agendas.

## The way forward

The current carbon market model undermines land-use practices, limits contributions to planetary health, and further marginalises particular groups of people, including Indigenous Peoples. This situation needs to change. The examples from Thailand, Australia, and Canada highlighted in this Personal View show both the challenges and opportunities going forward.

Ultimately, the interactions between Indigenous Peoples and carbon markets are complex and nuanced, and associated with the relevant legal and political circumstances in a respective region. The interactions will additionally vary based on levels of awareness, respect, trust, and accountability, as well as levels of inherent Indigenous decision-making power within a respective region. Currently, most carbon-related mechanisms are clearly operating in a colonial way (ie, perpetuating a new form of colonialism), risking ongoing and direct harm to Indigenous communities and the planet. Some other Indigenous-led carbon credit projects are showcasing the importance of self-determination and the ability of Indigenous Peoples to be leaders within the space if they so choose. Therefore, carbon markets could be seen as a potential revenue source for Indigenous-led land management when verification processes consider and respect diverse ways of knowing (eg, Indigenous traditional knowledges).

Many Indigenous Peoples also strongly push back at the notion that the land should have a monetary value, with the concept of carbon markets themselves showing a colonial de-valuing and disconnection from nature. This worldview of humans being disconnected from the land they walk on perpetuates climate solutions (ie, carbon markets) that are also themselves devoid of connections to the land. Indigenous views and perspectives must therefore be respected and honoured to avoid further harm and to ensure Indigenous rights are upheld.

For entities seeking to work with Indigenous nations that do have an interest in carbon market participation, new codesign carbon marketplaces are needed that prioritise Indigenous values, with appropriate monitors to prevent homogenisation of the market (ie, little to no flexibility for bespoke preferences), and ensure buyer adherence to Indigenous principles. To value traditional knowledges and optimise land and soil conservation, carbon markets and related mechanisms should also equally value Indigenous-based indicators of land management, and their rights to define such indicators. Several elements of any carbon market initiative must be made standard to ensure the chance of long-term planetary health for all, including: free, prior, and informed consent; respect for Indigenous self-determination and land tenure rights; recognition of Indigenous knowledges; recognition of the human right to a clean, healthy, and sustainable environment; and alignment of carbon market structures with the UN Declaration on the Rights of Indigenous Peoples.

Indigenous Peoples have been stated to be a key determinant of planetary health, with the health of the planet being intrinsically tied to the wellbeing of Indigenous Peoples.^27^

“When Indigenous Peoples have their Land, culture, and sovereignty, they are more likely to have greater wellbeing. Thus, they will continue to sustainably care for…the worlds old-growth forests and the most biodiverse regions on the planet.”^27^

Whether carbon markets are a successful strategy for non-Indigenous peoples in the context of environmental management is less contested. From an Indigenous perspective, whether the orientation towards market-based solutions of nature will work to support the planet could well be contested. Orienting the hearts and minds of non-Indigenous peoples to appreciate themselves as being interconnected within nature will, in our view, not come through monetary-based policies.

“…it became clear that Mother Earth['s health] is dependent on the human capacity to understand interconnectedness as a basic and fundamental reality.”^27^

### Contributors

## Declaration of interests

We declare no competing interests.
